# *KRAS* mutation testing in the treatment of metastatic colorectal cancer with anti-EGFR therapies

**DOI:** 10.3747/co.v17is1.614

**Published:** 2010-07

**Authors:** D. Soulières, W. Greer, Anthony M. Magliocco, D. Huntsman, S. Young, M.-S. Tsao, S. Kamel-Reid

**Affiliations:** * Centre Hospitalier de l’Université de Montréal, Montreal, Quebec; † Department of Pathology, Dalhousie University, Halifax, Nova Scotia; ‡ Departments of Oncology, Pathology and Laboratory Medicine, University of Calgary; Tom Baker Cancer Centre; § BC Cancer Agency, Vancouver, BC; || University Health Network and The University of Toronto

**Keywords:** KRAS, EGFR, colorectal carcinoma, cetuximab, panitumumab, genetic testing methods

## Abstract

Survival of patients with metastatic CRC (mCRC) has improved steadily over the past several decades, due largely to the development of new combinations of standard chemotherapy, as well as to the introduction of new targeted therapies. Among the available targeted therapies are two monoclonal antibodies that target the epidermal growth factor receptor (EGFR) – cetuximab and panitumumab – which have demonstrated efficacy in the treatment of mCRC. These therapies are associated with a unique set of toxicities and costs, prompting the need for tools to select patients who are most likely to derive a benefit from them. Mutations in the *KRAS* oncogene have consistently been shown to predict non-response to cetuximab and panitumumab. The role of KRAS as a marker of efficacy of anti-EGFR therapies is reviewed.

## 1. INTRODUCTION

Colorectal cancer (CRC) affects more than 21,000 Canadians each year, and is the second leading cause of death from cancer among Canadian men and women^1^. Survival of patients with metastatic CRC (mCRC) has improved steadily over the past several decades, due largely to the development of new combinations of standard chemotherapy such as 5-fluorouracil, irinotecan, and oxaliplatin, as well as to the introduction of new targeted therapies. Among the available targeted therapies are two monoclonal antibodies that target the epidermal growth factor receptor (EGFR) – cetuximab and panitumumab – which have clearly demonstrated efficacy in the treatment of mCRC. However, the introduction of these therapies has also introduced a unique set of toxicities and increased costs^2^, prompting the need for tools to select the patients who are most likely to benefit from these therapies.

There has recently been heightened interest in the relevance of several biomarkers for the selection of patients who will benefit from EGFR-targeted therapies for the treatment of CRC and other EGFR-associated cancers. In particular, mutations in the *KRAS* oncogene have consistently been shown to predict nonresponse to cetuximab and panitumumab. This marker is of particular importance, given the prevalence of *KRAS* mutations among patients with CRC; up to half of patients with CRC are found to have the mutant version of the gene^3^–^6^.

### 1.1 *KRAS* and the EGFR pathway

**Figure 1:**
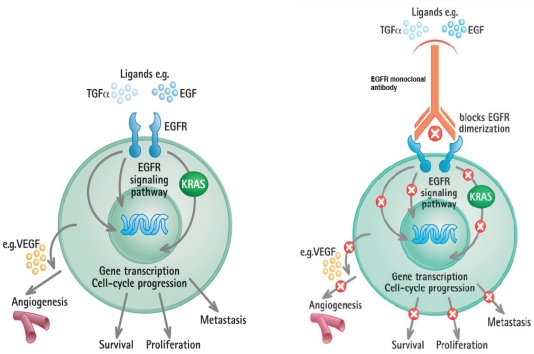
Left: the EGFR pathway; Right: inhibition of the EGFR pathway by the EGFR monoclonal antibodies

*KRAS* is a signal transducer downstream of tyrosine kinase receptors including EGFR – a complex signaling cascade involved in the development and progression of cancer. The EGFR pathway is activated by the binding of the cell-surface EGFR/HER family receptors to their ligands, such as transforming growth factor alpha (TGF- α) and EGF. This leads to activation of genes that regulate cell cycle progression, tumor cell survival, metastases and angiogenesis (Fig. 1). Monoclonal antibodies against EGFR, such as cetuximab and panitumumab, block the receptor signaling and its downstream events, including those mediated by *KRAS*.

Upon stimulation of the EGFR, wild-type *KRAS* is active for a short period and the signaling activities to the downstream RAF/mitogen-activated protein kinase (MAPK)/extracellular signal-related kinase (ERK) pathway are tightly controlled. Mutated *KRAS* protein becomes constitutively activated, thereby making the cascade independent of upstream signaling by tyrosine kinase receptors such as the EGFR. Therefore, blocking of EGFR with cetuximab or panitumumab may not affect downstream events. Mutations within the *KRAS* gene resulting in constitutive protein activity are found in approximately 30% to 50% of all CRCs^3^–^6^.

### 1.2 The role of *KRAS* and BRAF as markers of efficacy of the anti-EGFR therapy

#### 1.2.1 KRAS

As reviewed by Fakih and Wong in this supplement, the efficacy of the anti-EGFR antibodies cetuximab and panitumumab in the treatment of mCRC has consistently been shown to rely on the *KRAS* status of the tumor (Tables 1 and 2). Post hoc analyses of both randomized and single-arm trials of cetuximab or panitumumab have demonstrated that these monoclonal antibodies are only effective against tumors with wild-type *KRAS*, while patients with *KRAS* mutations in codon 12 or 13 do not derive any benefit from these treatments^3^,^4^,^6^–^11^.

The first study to provide conclusive data showing the relationship between *KRAS* status and the efficacy of the monoclonal anti-EGFR antibody panitumumab was Amado’s analysis of tumors from 427 mCRC patients who were randomly assigned to treatment with panitumumab or best supportive care (BSC)^6^. Treatment response and improvement in progression-free survival (PFS) with panitumumab monotherapy were both limited to patients with wild-type *KRAS*. Of the 84 panitumumab treated patients with *KRAS* mutations, none responded to the treatment. In contrast, 21 of 124 antibody-treated patients with wild-type *KRAS* tumors experienced a partial response. Among patients with wild-type *KRAS*, PFS was significantly improved with panitumumab compared with BSC alone (HR 0.45; 95% CI 0.34–0.59; median PFS 12.3 weeks for panitumumab vs. 7.3 weeks for BSC), while no benefit was observed among those with mutant *KRAS* (HR 0.99; 95% CI 0.73–1.36; median PFS of 7.4 weeks for panitumumab vs. 7.3 weeks for BSC).

Similar results have been demonstrated with cetuximab. In a retrospective analysis of 540 mutation assessable patients in the CRYSTAL (Cetuximab Combined with Irinotecan in First-Line Therapy for Metastatic Colorectal Cancer) trial, *KRAS* mutations were identified in 35.6%^4^. For patients with wild-type *KRAS*, the addition of cetuximab to folinic acid, fluorouracil, and irinotecan (FOLFIRI) improved both PFS (9.9 vs. 8.7 months; HR 0.68; P=0.017) and response rate (59.3% vs. 43.2%; P=0.0025). In contrast, for patients with *KRAS* mutations, treatment with cetuximab did not significantly improve either PFS (7.6 vs. 8.1 months; HR 1.07; P=0.47) or response rate (40.2% vs. 36.2%; P=0.46) in comparison with FOLFIRI alone. In the OPUS (Oxaliplatin and Cetuximab for First-Line Treatment of Metastatic Colorectal Cancer) study, patients were treated with first-line infused fluorouracil, folinic acid, and oxaliplatin (FOLFOX) with or without cetuximab^3^. Response rate and PFS were both significantly improved in patients treated with cetuximab; however, these benefits were limited to those with wild-type *KRAS* tumors, and patients with mutated *KRAS* receiving cetuximab demonstrated poorer outcomes than those receiving FOLFOX alone.

The phase III CO.17 trial, conducted by the National Cancer Institute of Canada Clinical Trials Group (NCIC CTG) in collaboration with the Australasian Gastro-Intestinal Trials Group (AGITG), examined the effect of cetuximab on survival among patients with advanced CRC in whom all chemotherapy had failed and for whom no other standard anticancer therapy was available^12^. Although cetuximab used alone in the third-line setting improved overall survival and PFS and preserved quality of life to a better degree than BSC alone, resistance to cetuximab was high, with more than half of the cetuximab-treated patients showing progression at the first assessment of disease response. With the accumulating evidence demonstrating the ineffectiveness of anti-EGFR in patients with *KRAS* mutations, the study group undertook correlative analyses to determine whether the mutation status of the *KRAS* gene modified the effect of cetuximab on the overall survival (OS) and PFS in the CO.17 patient population^9^. A total of 394 tumor samples – 198 from the cetuximab group and 196 from the BSC group – were available for *KRAS* analysis, accounting for 68.9% of the original study population. *KRAS* mutations were detected in 40.9% and 42.3% of tumors from the cetuximab and BSC groups, respectively. Among patients with wild-type *KRAS*, median overall survival was significantly longer in the cetuximab group (9.5 months) than in the BSC group (4.8 months), with one-year overall survival rates of 28.3% and 20.1%, respectively (HR 0.55, 95% CI 0.41–0.74, P<0.001). However, among patients with *KRAS* mutations, overall survival was not improved with cetuximab, with a median survival of 4.5 months vs. 4.6 months with BSC alone, and one-year overall survival rates of 13.2% and 19.6%, respectively (HR 0.98, 95% CI 0.70–1.37, p=0.89). These results were instrumental in defining the indication for the monoclonal antibody EGFR inhibitors in North America.

#### 1.2.2 BRAF

In the absence of *KRAS* mutations, resistance to anti-EGFR therapies may occur as a result of mutations in other signaling molecules in the RAS-RAF-MAPK pathway. Recent retrospective analyses of mCRC patients treated with cetuximab or panitumumab have shown that BRAF mutations, which are exclusive from *KRAS* mutations, occur in approximately14% of patients and are also associated with a lack of response to anti-EGFR therapy^13^,^14^. BRAF mutations have also been associated with significantly shorter PFS^13^,^14^ and overall survival^13^ in patients with mCRC.

## 2. METHODS FOR KRAS TESTING

### 2.1 Guidelines for testing

In Canada, panitumumab is currently restricted to the treatment of EGFR-expressing mCRC with non-mutated (wild-type) *KRAS*^15^, while KRAS status is not specified in the indication for cetuximab^16^. However, with the knowledge that patients with mCRC who harbour *KRAS* mutations do not derive any benefit from treatment with EGFR-targeting monoclonal antibodies in the first-, second-, or third-line settings, Cancer Care Ontario (CCO) recommends the two clinically available EGFR inhibitors, cetuximab and panitumumab, be used “for the treatment of patients with advanced CRC after failure of standard chemotherapy and whose tumors have tested negative for *KRAS* gene mutations”^17^. CCO has also recently approved the use of cetuximab in combination with irinotecan for the third-line treatment of mCRC only in the presence of tumors with the non-mutated KRAS oncogene.

The American Society of Clinical Oncology (ASCO) recently also released a provisional clinical opinion recommending that patients with mCRC who are candidates for treatment with cetuximab or panitumumab undergo tumor testing for *KRAS* mutations in a CLIA-accredited laboratory^18^. The updated National Comprehensive Cancer Network (NCCN) clinical practice guidelines for colon cancer and rectal cancer also recommend testing for *KRAS* gene mutations, stipulating that only patients with wild-type *KRAS* genes should receive treatment with cetuximab or panitumumab^19^,^20^.

However, BRAF testing is currently not a requirement for treatment with an EGFR inhibitor.

### 2.2 How to test?

By screening patients with mCRC for *KRAS* tumor status prior to initiating treatment with an anti-EGFR monoclonal antibody, unnecessary toxicity and costs can be avoided for patients who are unlikely to respond. However, there are logistical challenges in testing tumors from mCRC patients, as well as questions surrounding specimen selection, and selection of the appropriate assay.

### 2.3 Specimen selection

The most readily available clinical specimens for mutational analysis are typically formalin-fixed, paraffin-embedded (FFPE) tissue blocks. Until recently, formalin-fixed samples were considered to be of low quality and yield for DNA testing^21^; however, improvements in techniques have enhanced the ability to use DNA from FFPE tissue^22^. Because the fixation process damages DNA and can potentially introduce artificial mutations in conventional PCR processes, sufficient cellular material is necessary for analysis^23^. DNA from surrounding reactive cells, such as fibroblasts, leukocytes, or endothelial cells, can also potentially compete with mutant DNA in amplification reactions and introduce errors in testing. Tumor cell enrichment by micro- or macro-dissection or selective sampling of the paraffin block by needle core may increase the sensitivity of mutation testing, but care must be taken with these procedures to ensure that sufficient DNA is available for amplification^22^ and contamination of normal DNA with mutant DNA does not occur.

Based on current knowledge, the most appropriate specimens for *KRAS* mutation testing may be obtained from the primary tumor^24^. However, an estimated 20% of patients will present with metastatic disease and, therefore, lack tissues from the primary tumor^24^. In these patients, *KRAS* testing may be performed using material from the metastatic tumor^24^. Data comparing *KRAS* status in primary and metastatic tumors are limited and have had inconsistent results^25^–^28^. However, in a recent Italian study of 48 patients with mCRC, DNA sequencing revealed an overall concordance of *KRAS* mutational status between primary tumors and metastases in 92% of patients, suggesting that evaluation of *KRAS* status can be performed in either the primary tumor or metastatic sites^29^.

### 2.4 Assay selection

Several mutation detection procedures have been described, all of which are based on the polymerase chain reaction (PCR) (Table 3)^22^. Generally, selection of the best technique for use is based on the sample size, desired level of sensitivity and DNA quality and quantity available.

### 2.5 Direct sequencing of PCR products

Direct sequencing of PCR products detects all mutations in amplified DNA sequences, and this is currently the most commonly used method for *KRAS* testing^30^. However, this method requires mutant copies to have a minimum concentration of 20% to 50% that of any accompanying wild-type sequences, and will therefore miss mutations that may be present at a lower level 21,31. Because of the low sensitivity and the expense of direct sequencing, more sensitive and specific assays have been developed to assess *KRAS* in clinical samples, including methods that employ restriction fragmentation length polymorphism (RFLP), allele-specific oligonucleotide (ASO) hybridization, high resolution melting analysis (HRMA), and amplification refractory mutation systems (ARMS).

### 2.6 RFLP

Whereas gene sequencing compares the sequence of the sample gene with the normal sequence of the gene, nucleotide by nucleotide, RFLP methods detect differences between mutant and wild-type DNA by their susceptibility to digestion by restriction enzymes. Restriction enzymes can be selected to recognize a defined sequence which is present only in the mutated or non-mutated DNA. Knowing what specific size the digested fragment should be in mutated vs. non-mutated DNA allows one to identify if a mutation is present or not. These amplified mutant copies can then be detected by gel or capillary electrophoresis^32^. While highly sensitive, this method is also very complex, requiring tight control of PCR and digestion conditions to avoid replication errors and artificial mutations. Furthermore, when a mutation is detected by this methodology the specific nucleotide change cannot be identified. If the specific mutation identity is required (this is not currently necessary for clinical utility), then direct sequencing can be used after mutation identification by RFLP.

### 2.7 Allele-specific oligonucleotide hybridization

Short segments of synthetically produced DNA (oligonucleotides) can be used to detect mutations in a gene segment. The oligonucleotides are complementary to a corresponding segment of the gene under investigation, hybridizing completely with the wild-type sequence or to one of the possible mutations. A single base mismatch caused by a mutation reduces the melting point temperature of the double-stranded hybrid^33^. The difference in melting points between matched and mismatched sequences can be used to detect single-base mismatches between wild-type and mutant sequences^34^.

Finding rare mutant alleles in a DNA mixture can be challenging however, particularly in samples containing high levels of normal alleles. Therefore, if a sample has a low tumor burden, this may not be the best approach. This technique is also expensive, requiring specialized equipment and software for analysis^35^.

### 2.8 High resolution melting analysis

The presence of a mutation disrupts the affinity of two DNA chains, causing them to bind with less energy and become more easily separated by heat. High resolution melting analysis (HRMA) is performed following PCR, and measures differences in melting point temperatures between matched and mismatched double stranded DNA, caused by polymorphisms or somatic mutations^35^. HRMA has a high sensitivity, and is also inexpensive and fast. However, because any DNA alteration can produce an abnormal melting point curve, abnormal curves need to be confirmed by sequencing, which increases turnaround time and expense, reducing HRMA’s advantage over direct sequencing alone. HRMA may therefore be useful for rapid screening; however the need for confirmation by sequencing may limit its utility in the clinical setting.

### 2.9 Amplification refractory mutation system

The amplification refractory mutation system (ARMS) – also known as allele-specific PCR or PCR amplification of specific alleles – utilizes a PCR primer which is designed to discriminate among templates that differ by a single nucleotide residue. The ARMS primer can be designed to amplify a specific allele of a multi-allelic system while remaining refractory to amplification of another allele that may differ by as little as a single base. ARMS is able to detect directly the presence of *KRAS* mutations in heterogeneous specimens at a low allelic concentration (1%) without the need for confirmation by direct sequencing. A drawback of ARMS is that it is only able to detect known mutations; separate reactions are required for each mutation, thus requiring more DNA material. However, because the amplification step and the diagnostic steps are combined, this may prove to be a time-efficient and practical method for routine diagnosis of *KRAS* mutations^22^.

### 2.10 Standardization of *KRAS* testing in colorectal cancer

Because of the potential for variability among the different testing methods, a thorough analytical validation of testing methods, together with a high standard of quality assurance are critical for accurate, reliable testing of *KRAS* mutations in clinical practice^24^. In Canada, Health Canada has approved the use of the TheraScreen K-RAS testing kit (DxS, Manchester, United Kingdom), which combines ARMS with a real time PCR technology. In the United States, there is currently no FDA-approved test for *KRAS* testing, and testing can be performed using laboratory-developed tests, provided that the laboratory is accredited by the College of American Pathologists (CAP) and the test has been appropriately validated.^36^ To date, one published study has evaluated the concordance between different methods for *KRAS* mutation testing^37^. Four commercially available assays were used to assess seven common mutations of the *KRAS* gene in codons 12 and 13 in 40 colorectal tumor samples, with direct sequencing used as a reference. Two of the allele-specific PCR-based methods and one PCR/direct sequencing method demonstrated high to good agreement with direct sequencing, whereas an oligonucleotide hybridization method showed poor agreement.

## 3. RESULTS

The authors of the present article conducted a small study involving six Canadian laboratories to compare the accuracy and sensitivity of three methods of *KRAS* mutation analysis – the TheraScreen K-RAS testing kit only (1 laboratory), the TheraScreen K-RAS testing kit in combination with direct sequencing (2 laboratories), the TheraScreen K-RAS testing kit in combination with direct sequencing and RFLP (1 laboratory), and RFLP plus sequencing (2 laboratories). In the first phase of the study, 10 DNA samples were extracted from seven *KRAS* mutant (positive) cell lines containing approximately 50–100% mutant cells. In the second phase, dilutions were created from each of the seven positive cell lines (approximately 10–40% mutant cells). To assess the ability of the laboratory to extract DNA from paraffin and the resulting specificity, accuracy and sensitivity of *KRAS* mutation testing on such samples, 8–10 samples were extracted from paraffin blocks for the third phase of the study. For each phase, *KRAS*-negative cell lines were used for comparison.

All of the labs were able to detect *KRAS* mutations in samples derived from cell lines containing 50–100% mutant *KRAS* cells as well as from diluted cells lines containing 10–40% mutant *KRAS* cells. However, two of the labs experienced some difficulty interpreting two samples from the diluted cell lines when using sequencing methodology; this is probably due to the limits of sensitivity of sequencing.

Concordant results were achieved with five of the eight samples extracted from paraffin blocks. Inconsistent results with RFLP plus sequencing were seen in one lab, which was later discovered to be due to a mix-up of the samples (Lab 5, samples 3 and 4, Table 4). Discordant results were reported for three of the eight samples. In sample four, results were not concordant as the sample had a low level *KRAS* mutation requiring a very sensitive assay, prompting the question of what the sensitivity cutoff of an assay should be. In sample six, two labs reported inconclusive results using the TheraScreen test, suggesting that labs reporting inconclusive results with this test should reconsider their delta-Ct cutoff criteria, optimize their assay, or use another method to verify the results. In sample eight, most of the labs had some difficulty in interpreting the mutation status due to the limited tumor area on the slides and a non-formalin based fixation method, resulting in low DNA yield and poor DNA quality. The labs that participated are all well-experienced in performing complex genetic analyses on various sample types. These results thus point to some of the challenges of *KRAS* testing in poor quality samples.

## 4. CONCLUSIONS

In all clinical trials, anti-EGFR therapies have been consistently ineffective in mCRC patients with *KRAS* mutations. Targeting these therapies based on *KRAS* status will not only spare patients ineffective and toxic therapies, but will also greatly reduce unnecessary costs. The economic implications of customizing anti-EGFR therapy based on *KRAS* status was recently evaluated by Shakaran et al., using estimated incidence rates for new mCRC cases in the United States^38^. Based on an annual incidence of 29,762 new cases of mCRC cases, the cost of upfront *KRAS* testing was calculated at $13 million ($452/patient). By treating only the estimated 64.4% of patients with wild-type *KRAS*, net savings were estimated to be $740 million in the U.S. Although cetuximab is used more commonly in the third-line setting where treatment duration is shorter, targeting treatment based on *KRAS* status is likely to result in cost savings across all lines of therapy^38^.

Testing techniques need to be standardized and validated externally as well as internally. Cell line materials provided the most accurate results, while paraffin-embedded tissue may be somewhat more problematic, especially if suboptimal.

The role of the pathologist is very important in *KRAS* testing. The pathologist is responsible for choosing the most appropriate tissue block to be tested, evaluating the tumor content of the tissue block, and ensuring that it is adequate (tissue size, degree of tumor involvement) by assessing the H&E-stained section of the tissue area and marking the area with adequate tumor density – preferably >70% carcinoma cells. Testing should be performed by an accredited and licensed testing lab that conforms to quality guidelines for *KRAS* testing^24^ and routinely participates in proficiency testing (e.g. the College of American Pathologists [CAP]).

We propose an algorithmic approach to *KRAS* testing, where laboratory professionals (pathologists, geneticists) have access to multiple methods wherever possible, especially when assessing suboptimal material. We have found that for small samples with degraded DNA, Sanger sequencing is often still the best method for mutation detection.

Regardless of the testing method used to determine *KRAS* status in patients with mCRC, the goal is for a sensitive and specific technique that has been standardized and validated externally and internally. As noted above, CAP currently has a proficiency challenge available for labs so they can assess their ability to test for *KRAS* mutations.

The anti-EGFR monoclonal antibody therapies are currently approved for the treatment of mCRC in the third-line setting. However, it may be several weeks before results of *KRAS* testing are obtained, which can be a long wait for a patient with advanced disease who requires treatment. Depending on the availability of funding, it would be optimal for KRAS testing to begin immediately following a diagnosis of metastatic disease; currently however, testing can only be undertaken when the Oncologist is considering third-line therapy.

## Figures and Tables

**Table I.** Randomized clinical trial evidence on the relationship of KRAS mutation status to efficacy of anti-EGFR monoclonal antibodies in patients with metastatic colorectal cancer

			***KRAS* Wild-type**		***KRAS* Mutated**	

*Study and Population*	*Treatments by Arm*	*Variable*	*Antibody Arm*	*Control Arm*	*Antibody Arm*	*Control Arm*
van Cutsem et al, 2008^4^; CRYSTAL trial of first-line therapy	FOLFIRI ± cetuximab	n	172	176	105	87
		RR (%)	59.3	43.2	36.2	40.2
		95% CI	51.6–66.7	35.8–50.9	27.0–46.2	29.9–51.3
		P	0.0025		0.46	
		Median PFS (mo)	9.9	8.7	7.6	8.1
		HR	0.68		1.07	
		P	0.017		0.47	
Bokemeyer et al, 2009^39^; OPUS trial of first-line therapy	FOLFOX - cetuximab	n	61	73	52	47
		RR (%)	60.7	37.0	32.7	48.9
		95% CI	47.3–72.9	26.0–49.1	20.3–47.1	34.1–63.9
		P	0.011		0.106	
		OR	2.54		0.51	
		95% CI	1.24–5.23		0.22–1.15	
		Median PFS (mo)	7.7	7.2	5.5	8.6
		HR	0.57		1.83	
		P	0.016		0.0192	
Punt et al, 2008^11^; CAIRO2 trial of first-line therapy	(Capecitabine + oxaliplatin + bevacizumab) ± cetuximab	n	153	152	93	103
		Median PFS (mo)	10.5	10.7	8.6	12.5
		P	0.10		0.43	
		Median OS (mo)	22.2	23.0	19.1	24.9
		P	0.49		0.35	
Amado et al, 2008^6^; Chemotherapy-refractory disease	Panitumumab *v* best supportive care	n	124	119	84	100
		RR (%)	17	0	0	0
		Median PFS (wks)	12.3	7.3	7.4	7.3
		HR	0.45		0.99	
		95% CI	0.34–0.59		0.73–1.36	
Karapetis et al, 2008^9^; second- or subsequent-line therapy	Cetuximab *v* best supportive care	n	117	113	81	83
		RR (%)	12.8	0	1.2	0
		Median PFS (mo)	3.7	1.9	1.8	1.8
		HR	0.40		0.99	
		95% CI	0.30–0.54		0.73–1.36	
		P	<0.001		0.96	
		Median OS (mo)	9.5	4.8	4.5	4.6
		P	0.01 (for interaction, *KRAS* mutation status and treatment arm)			
		OS at 1 yr (%)	28.3	20.1	13.2	19.6
		HR (death)	0.55		0.98	
		95% CI	0.41–0.74		0.70–1.37	
		P	<0.001		0.89	

EGFR = Epidermal growth factor receptor; HR = hazard ratio; OR = odds ratio; PFS = progression-free survival; FOLFIRI = folinic acid, fluorouracil, and irinotecan; FOLFOX = folinic acid, fluorouracil, and oxaliplatin; CRYSTAL = Cetuximab Combined With Irinotecan in First-Line Therapy for Metastatic Colorectal Cancer; OPUS = Oxaliplatin and Cetuximab in First-Line Treatment of mCRC; CAIRO2, Capecitabine, Irinotecan, and Oxaliplatin in Advanced Colorectal Cancer; RR = Risk reduction

Adapted with permission: BlueCross BlueShield Association. Technology Evaluation Center. KRAS Mutations and Epidermal Growth Factor Receptor Inhibitor Therapy in Metastatic Colorectal Cancer TEC Assessments 2008; volume 23, tab 6. Copyright © 2008, BlueCross BlueShield Association.

**Table II.** Single-arm studies of treatment of metastatic colorectal cancer with anti-EGFR monoclonal antibodies and KRAS mutational status

*Study and Population*	*Treatments by Arm*	*Variable*	*KRAS Wild-type*	*KRAS Mutated*
Lievre et al, 2008^10^; second-line therapy	Cetuximab	n	65	24
		RR	40	0
		P	0.001	
		PFS, weeks	31.4	10.1
		95% CI	19.4 to 36	8 to 16
		P	0.0001	
		OS, months	14.3	10.1
		95% CI	9.4 to 20	5.1 to 13
		P	0.026	
De Roock et al, 2008^8^	Cetuximab alone v with irinotecan	n	57	46
		RR	41	0
		P (cetuximab vs. irinotecan)	0.000001	
		P (cetuximab alone)	0.126	
		PFS cetuximab vs. irinotecan (weeks)	34	12
		95% CI	28.5 to 40.0	5.4 to 18.7
		P	0.016	
		PFS cetuximab (weeks)	12	12
		95% CI	4.2 to 20.0	7.0 to 17.0
		P	0.351	
		OS cetuximab. irinotecan (weeks)	44.7	27.3
		95% CI	28.4 to 61.0	9.5 to 45.0
		P	0.003	
		OS (weeks)	27	25.3
		95% CI	8.9 to 45.1	0.0 to 70.0
		P	0.33	
Khambata-Ford et al, 2007^7^	Cetuximab; second-or third-line treatment	n	50	30
		RR (%)	10	0
Di Fiore et al, 20077	Cetuximab plus chemotherapy	n	43	16
		RR (%)	28	0
Benvenuti et al, 200740	Panitumumab or cetuximab, or cetuximab plus chemotherapy	n	32	16
		RR (%)	31	6

EGFR = epidermal growth factor receptor; PFS = progression-free survival; OS = overall survival.

Adapted with permission: BlueCross BlueShield Association. Technology Evaluation Center. KRAS Mutations and Epidermal Growth Factor Receptor Inhibitor Therapy in Metastatic Colorectal Cancer TEC Assessments 2008; volume 23, tab 6. Copyright © 2008, BlueCross BlueShield Association.

**Table III.** Methods for analyzing *KRAS* mutations^22^,^41^

*Method*	*Principle*	*Sensitivity (MT/WT, %)*	*Turnaround*	*Advantages*	*Disadvantages*
Direct sequencing	Non-mutation-specific determination of test case nucleotide sequence and comparison with normal sequence	15–25	Slow (4 days to 2 weeks from paraffin)	Gold standard Detects all possible mutations	Poorly quantitative Insensitive; Labour intensive Open PCR system requires strict control to prevent contamination
RFLP	Mutation presence induces or eliminates specific sites where DNA-targeting enzymes insert cuts in DNA	1	Slow (4 days to 2 weeks from paraffin)	Requires no specialized equipment, inexpensive	Often requires confirmation by sequencing Does not identify specific mutation Non-quantitative
Allele-specific probe	Polymerase chain reaction/selective detection	10	Rapid (<2 days from paraffin)	Rapid turnaround	Relatively low sensitivity
High resolution melting analysis, confirmed by direct sequencing	Sequences with mutations hybridize at different, fixed temperatures	5	Slow (4 days to 2 weeks from paraffin)	Can screen for mutations prior to sequencing	Complicated Requires sequencing confirmation Considerable manual input required
Amplification refractory mutation system (ARMS)	Mutation specific polymerase chain reaction/detection	1	Rapid (<2 days from paraffin)	High sensitivity Rapid turnaround	Detects only single specific mutation per reaction Requires specially engineered primer/probe
TheraScreen™ KRAS testing kit (DxS, Manchester, United Kingdom)	Combination of ARMS and real-time PCR technology	1–5%	Rapid (2 days)	High sensitivity Rapid turnaround Closed PCR system eliminates risk of contamination	Detects only the most common mutations Requires more tissue for analysis than other methods Very Expensive
				Available as a commercial kit	
Pyrosequencing	Detection and measurement of the amount of pyrophosphate released by DNA extension reaction	5–10	Rapid	Precise and reproducible allele quantification Allows sequencing of relatively small PCR products (useful for degraded DNA samples).	Short reading length for sequences used Open PCR system requires strict control to prevent contamination

MT = Mutant; WT = Wild-type

**Table IV.** *KRAS* results of 8 mCRC tumor samples extracted from paraffin blocks: concordance among six testing laboratories

	*Reference Lab (Lab1)*							
*Sample ID*	*DxS*	*Sequencing*	*FAM-labeled RFLP*	*Lab 2*	*Lab 3*	*Lab 4*	*Lab 5*	*Lab 6*
1	Gly12Asp	Gly12Asp	Codon 12 +	√	√	√	√	√
2	Gly12Val	Gly12Val	Codon 12 +	√	√	√	√	√
3	Wild Type	Wild Type	Wild Type	√	√	√	RFLP:+ Seq: −	√
4	Gly12Asp? Δct 7.6	Wild Type	Codon 12 +? Low fluor	wt	wt	wt	wt	DxS Gly12Asp? Seq: wt
5	Gly13Asp	Gly13Asp	Not Done	√	√	√	√	√
6	Wild Type	Wild Type	Wild Type	√	DxS: ? G13Asp	√	√	DxS: ? Seq: wt
7	Gly12Asp	Gly12Asp	Codon 12 +	√	√	√	√	√
8	Not Done	Gly12Asp	Codon 12 +	√	Not determined	Need to repeat	RFLP: 12+ Seq: ?	No tumor

## References

[1] Canadian Cancer Society’s Steering Committee (2009). Canadian Cancer Statistics 2009.

[2] Schrag D (2004). The price tag on progress: chemotherapy for colorectal cancer. N Engl J Med.

[3] Bokemeyer C, Bondarenko I, Hartmann JT (2008). KRAS status and efficacy of first-line treatment of patients with metastatic colorectal cancer (mCRC) with FOLFOX with or without cetuximab: The OPUS experience. J Clin Oncol.

[4] Van Cutsem E, Lang I, D’haens G (2008). KRAS status and efficacy in the first-line treatment of patients with metastatic colorectal cancer (mCRC) treated with FOLFIRI with or without cetuximab: The CRYSTAL experience. J Clin Oncol.

[5] Khambata-Ford S, Garrett CR, Meropol NJ (2007). Expression of epiregulin and amphiregulin and K-ras mutation status predict disease control in metastatic colorectal cancer patients treated with cetuximab. J Clin Oncol.

[6] Amado RG, Wolf M, Peeters M (2008). Wild-type KRAS is required for panitumumab efficacy in patients with metastatic colorectal cancer. J Clin Oncol.

[7] Di Fiore F, Blanchard F, Charbonnier F (2007). Clinical relevance of KRAS mutation detection in metastatic colorectal cancer treated by Cetuximab plus chemotherapy. Br J Cancer.

[8] De Roock W, Piessevaux H, De Schutter J (2008). KRAS wild-type state predicts survival and is associated to early radiological response in metastatic colorectal cancer treated with cetuximab. Ann Oncol.

[9] Karapetis CS, Khambata-Ford S, Jonker DJ (2008). K-ras mutations and benefit from cetuximab in advanced colorectal cancer. N Engl J Med.

[10] Lièvre A, Bachet JB, Boige V (2008). KRAS mutations as an independent prognostic factor in patients with advanced colorectal cancer treated with cetuximab. J Clin Oncol.

[11] Punt CJ, Tol J, Rodenburg CJ (2008). Randomized phase III study of capecitabine, oxaliplatin, and bevacizumab with or without cetuximab in advanced colorectal cancer (ACC), the CAIRO2 study of the Dutch Colorectal Cancer Group (DCCG). J Clin Oncol.

[12] Jonker DJ, O’Callaghan CJ, Karapetis CS (2007). Cetuximab for the treatment of colorectal cancer. N Engl J Med.

[13] Di Nicolantonio F, Martini M, Molinari F (2008). Wild-type BRAF is required for response to panitumumab or cetuximab in metastatic colorectal cancer. J Clin Oncol.

[14] Ruzzo A, Cremolini C, Loupakis F (2009). Association of BRAF mutations and EGFR Intron-1 L/L genotype with resistance to cetuximab plus irinotecan treatment in KRAS wild-type metastatic colorectal cancer patients. J Clin Oncol.

[15] (2009). Vectibix™ (panitumumab) Product Monograph.

[16] (2009). Erbitux (cetuximab) Product Monograph.

[17] Jonker D, Biagi J, Haynes AE (2008). The use of epidermal growth factor receptor inhibitors in advanced colorectal cancer. Committee to Evaluate Drugs/CCO Special Advice Report #8.

[18] Allegra CJ, Jessup JM, Somerfield MR (2009). American Society of Clinical Oncology provisional clinical opinion: testing for KRAS gene mutations in patients with metastatic colorectal carcinoma to predict response to anti-epidermal growth factor receptor monoclonal antibody therapy. J Clin Oncol.

[19] National Comprehensive Cancer Network NCCN Clinical Practice Guidelines in Oncology: Colon CancerV.2.2009. Accessed at www.nccn.org/professionals/physician_gls/PDF/colon.pdf on November 30, 2009. 10.6004/jnccn.2009.0056 19755046

[20] National Comprehensive Cancer Network NCCN Clinical Practice Guidelines in Oncology: Rectal CancerV.2.2009. Accessed at www.nccn.org/professionals/physician_gls/PDF/rectal.pdf on November 30, 2009. 10.6004/jnccn.2009.0057 19755047

[21] Frayling IM (2002). Methods of molecular analysis: mutation detection in solid tumours. Mol Pathol.

[22] Jimeno A, Messersmith WA, Hirsch FR (2009). KRAS mutations and sensitivity to epidermal growth factor receptor inhibitors in colorectal cancer: practical application of patient selection. J Clin Oncol.

[23] Williams C, Pontén F, Moberg C (1999). A high frequency of sequence alterations is due to formalin fixation of archival specimens. Am J Pathol.

[24] van Krieken JH, Jung A, Kirchner T (2008). KRAS mutation testing for predicting response to anti-EGFR therapy for colorectal carcinoma: proposal for an European quality assurance program. Virchows Arch.

[25] Oudejans JJ, Slebos RJ, Zoetmulder FA (1991). Differential activation of ras genes by point mutation in human colon cancer with metastases to either lung or liver. Int J Cancer.

[26] Suchy B, Zietz C, Rabes HM (1992). K-ras point mutations in human colorectal carcinomas: relation to aneuploidy and metastasis. Int J Cancer.

[27] Al-Mulla F, Going JJ, Sowden ET (1998). Heterogeneity of mutant versus wild-type Ki-ras in primary and metastatic colorectal carcinomas, and association of codon-12 valine with early mortality. J Pathol.

[28] Tortola S, Steinert R, Hantschick M (2001). Discordance between K-ras mutations in bone marrow micrometastases and the primary tumor in colorectal cancer. J Clin Oncol.

[29] Artale S, Sartore-Bianchi A, Veronese SM (2008). Mutations of KRAS and BRAF in primary and matched metastatic sites of colorectal cancer. J Clin Oncol.

[30] Nollau P, Wagener C (1997). Methods for detection of point mutations: performance and quality assessment. IFCC Scientific Division, Committee on Molecular Biology Techniques. Clin Chem.

[31] Gallegos Ruiz MI, Floor K, Rijmen F (2007). EGFR and K-ras mutation analysis in non-small cell lung cancer: comparison of paraffin embedded versus frozen specimens. Cell Oncol.

[32] Levi S, Urbano-Ispizua A, Gill R (1991). Multiple K-ras codon 12 mutations in cholangiocarcinomas demonstrated with a sensitive polymerase chain reaction technique. Cancer Res.

[33] Wallace RB, Johnson MJ, Hirose T (1981). The use of synthetic oligonucleotides as hybridization probes: II. Hybridization of oligonucleotides of mixed sequence to rabbit beta-globin DNA. Nucleic Acids Res.

[34] Conner BJ, Reyes AA, Morin C (1983). Detection of sickle cell beta S-globin allele by hybridizationwith synthetic oligonucleotides. Proc Natl Acad Sci USA. PNAS.

[35] Gundry CN, Vandersteen JG, Reed GH (2003). Amplicon melting analysis with labeled primers: A closed-tube method for differentiating homozygotes and heterozygotes. Clin Chem.

[36] College of American Pathologists Perspectives on Emerging Technologies KRAS mutation testing for colorectal cancer 9 2009 http://www.cap.org/apps/docs/committees/technology/KRAS.pdf Accessed November 30, 2009.

[37] Juan T, Suggs S, Wolf M A comparability study of 4 commercial KRAS tests [abstract #1811].

[38] Shankaran V, Bentrem DJ, Mulcahy MF Economic implications of Kras testing in metastatic colorectal cancer (mCRC) [Abstract 298].

[39] Bokemeyer C, Bondarenko I, Makhson A (2009). Fluorouracil, leucovorin, and oxaliplatin with and without cetuximab in the first-line treatment of metastatic colorectal cancer. J Clin Oncol.

[40] Benvenuti S, Sartore-Bianchi A, Di Nicolantonio F (2007). Oncogenic activation of the RAS/RAF signaling pathway impairs the response of metastatic colorectal cancers to anti-epidermal growth factor receptor antibody therapies. Cancer Res.

[41] Monzon FA, Ogino S, Hammond ME (2009). The role of KRAS mutation testing in the management of patients with metastatic colorectal cancer. Arch Pathol Lab Med.

